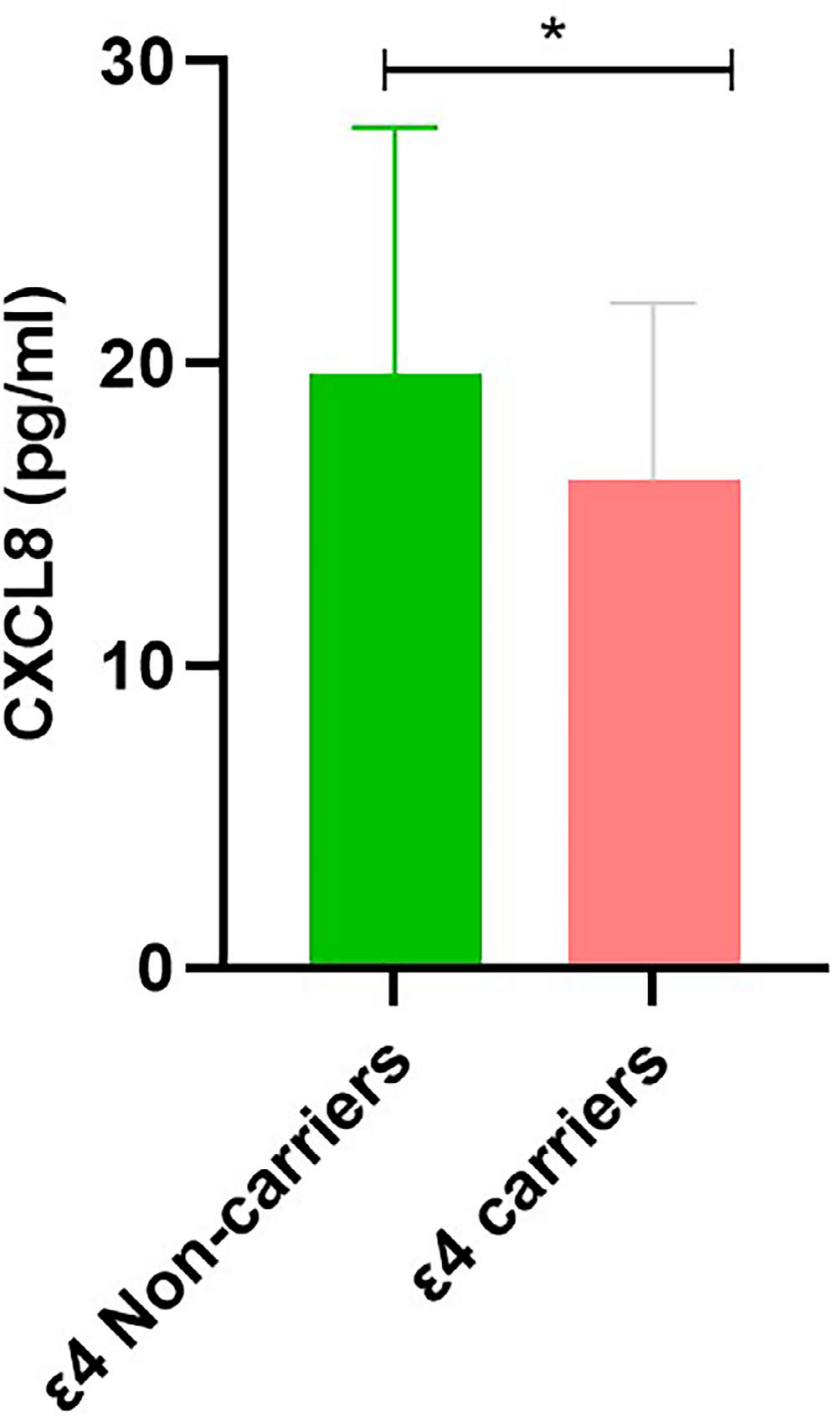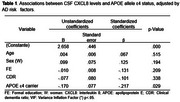# Relationship Between CXCL8 and APOE Ɛ4 allele in Cerebrospinal Fluid Samples from Brazilian Older Adults

**DOI:** 10.1002/alz70856_107390

**Published:** 2026-01-09

**Authors:** Júlia de Almeida Barreto, Ivonne Carolina Bolaños Burgos, Carolina Andrade Koehne, Flávia Carolina Lima Torres, Caio Mendes Ribeiro, Giovanna Correia Pereira Moro, Gabriela Tomé Oliveira Engelmann, Ana Caroline Nogueira‐Souza, Joice Coutinho de Alvarenga, Erika de Oliveira Hansen, Natália Silva Dias, Marco Aurelio Romano‐Silva, Debora Marques de Miranda, Jonas Jardim de Paula, Bernardo de Mattos Viana, Maria Aparecida Camargos Bicalho

**Affiliations:** ^1^ Cog‐Aging Research Group, Belo Horizonte, Minas Gerais, Brazil; ^2^ Neurotec R National Institute of Science and Technology (INCT‐Neurotec R), Faculty of Medicine, Universidade Federal de Minas Gerais (UFMG), Belo Horizonte, Minas Gerais, Brazil; ^3^ Undergraduate Medicine, Faculty of Medicine, Universidade Federal de Minas Gerais (UFMG), Belo Horizonte, Minas Gerais, Brazil; ^4^ Cog‐Aging Research Group, Universidade Federal de Minas Gerais (UFMG), Belo Horizonte, Minas Gerais, Brazil; ^5^ Cog‐Aging Research Group, Brazil, Belo Horizonte, Minas Gerais, Brazil; ^6^ Federal University of Minas Gerais, Belo Horizonte, Minas Gerais, Brazil; ^7^ Undergraduate medicine, Faculty of Medicine, Universidade Federal de Minas Gerais (UFMG), Belo Horizonte, Minas Gerais, Brazil; ^8^ Cog‐Aging Research Group, 31, Minas Gerais, Brazil; ^9^ Molecular Medicine Postgraduate Program, School of Medicine, Universidade Federal de Minas Gerais (UFMG), Belo Horizonte, Minas Gerais, Brazil; ^10^ Older Adult Psychiatry and Psychology Extension Program (PROEPSI), Faculty of Medicine, Universidade Federal de Minas Gerais (UFMG), Belo Horizonte, Minas Gerais, Brazil; ^11^ Department of Mental Health, Faculty of Medicine, Universidade Federal de Minas Gerais (UFMG), Belo Horizonte, Minas Gerais, Brazil; ^12^ Neurotec R National Institute of Science and Technology (INCT‐Neurotec R), Faculty of Medicine, Federal University of Minas Gerais, Belo Horizonte, Minas Gerais, Brazil; ^13^ Molecular Medicine Program, School of Medicine, Federal University of Minas Gerais, Belo Horizonte, Minas Gerais, Brazil; ^14^ Older Adult's Psychiatry and Psychology Extension Program (PROEPSI), School of Medicine, Universidade Federal de Minas Gerais (UFMG), Belo Horizonte, Minas Gerais, Brazil; ^15^ Department of Psychiatry, School of Medicine, Federal University of Minas Gerais, Belo Horizonte, Minas Gerais, Brazil; ^16^ Geriatrics and Gerontology Center Clinical Hospital of University of Minas Gerais, Belo Horizonte, Minas Gerais, Brazil; ^17^ Department of Internal Medicine, School of Medicine, Federal University of Minas gerais, Belo Horizonte, Minas Gerais, Brazil; ^18^ Sciences Applied to Adult Health Postgraduate Program, School of Medicine, Universidade Federal de Minas Gerais (UFMG), Belo Horizonte, Minas Gerais, Brazil

## Abstract

**Background:**

In Alzheimer's disease (AD), inflammation is a critical factor in disease progression. Among the cytokines involved in AD pathology, Interleukin 8 (CXCL8) has emerged as an important biomarker of neuroinflammation. Recent studies have identified a strong association between CXCL8 and the ApoE gene, particularly in carriers of the Ɛ4 allele, providing valuable insights into the role of these biomarkers in the pathophysiology of Alzheimer's disease.

**Objective:**

Determine the CXCL8 profile and its relationship with APOE allele Ɛ4 in a cohort of brazilian older adults.

**Method:**

One hundred and five older adults were enrolled and grouped based on APOE allele Ɛ4 status: APOE Ɛ4 carriers (*n* = 41) and non‐carriers (*n* = 64). Cerebrospinal fluid (CSF) samples were obtained via lumbar puncture, and levels of cytokines were assessed by the Luminex xMAP technique. Kendall correlation test between apoE allele Ɛ4 status and cytokines were performed. CXCL8 was prominently distinguished among the cytokines. Comparison between APOE Ɛ4 carriers and non‐carriers for CSF CXCL8 levels were explored using the Mann‐Whitney U‐test. Finally, to predict the influence of APOE Ɛ4 on CSF CXCL8 levels, linear regression models with logarithmic‐transformed variables were employed, adjusted for age, sex, formal education.

**Result:**

A significant negative correlation was found only between CSF CXCL8 levels and APOE Ɛ4 (r=‐0.193; *p* = 0.017). Furthermore, we observed a decreased CSF CXCL8 levels in Ɛ4 carriers compared to the non‐carriers group (U=1676.50; *p* = 0.017). We further explored these association using a linear regression model adjusted for sex, age, and formal education background, which revealed a negative significant association between CXCL8 levels and the presence of the Ɛ4 allele (ß= ‐ 0.217; *p* = 0.029).

**Conclusion:**

A decrease in CXCL8 levels was found to be associated with both APOE Ɛ4 allele in older brazilian adults, suggesting that CXCL8 may play a role in the pathology of neuroinflammation due to AD.